# Perspectives on team communication challenges in caring for children with medical complexity

**DOI:** 10.1186/s12913-021-06304-8

**Published:** 2021-04-01

**Authors:** Sherri Adams, Madison Beatty, Clara Moore, Arti Desai, Leah Bartlett, Erin Culbert, Eyal Cohen, Jennifer Stinson, Julia Orkin

**Affiliations:** 1 Division of Pediatric Medicine, SickKids, The Hospital for Sick Children, Toronto, Canada; 2 SickKids Research Institute, Toronto, Canada; 3 Lawrence S Bloomberg Faculty of Nursing, University of Toronto, Toronto, Canada; 4 Department of Pediatrics, University of Washington, Seattle, USA; 5 Royal Victoria Hospital, Barrie, Canada; 6 Credit Valley Hospital, Mississauga, Canada; 7 Department of Pediatrics, University of Toronto, Toronto, Canada; 8 Department of Anesthesia and Pain Medicine, Toronto, Canada

**Keywords:** Children with medical complexity, Communication challenges, Communication solutions, Shared decision making, Universal health record, Family centered care

## Abstract

**Background:**

Children with medical complexity (CMC) require the expertise of many care providers spanning different disciplines, institutions, and settings of care. This leads to duplicate health records, breakdowns in communication, and limited opportunities to provide comprehensive, collaborative care. The objectives of this study were to explore communication challenges and solutions/recommendations from multiple perspectives including (i) parents, (ii) HCPs – hospital and community providers, and (iii) teachers of CMC with a goal of informing patient care.

**Methods:**

This qualitative study utilized an interpretive description methodology. In-depth semi-structured interviews were conducted with parents and care team members of CMC. The interview guides targeted questions surrounding communication, coordination, access to information and roles in the health system. Interviews were conducted until thematic saturation was reached. Interviews were audio-recorded, transcribed verbatim, and coded and analyzed using thematic analysis.

**Results:**

Thirty-two individual interviews were conducted involving parents (*n* = 16) and care team members (*n* = 16). Interviews revealed 2 main themes and several associated subthemes (in parentheses): (1) Communication challenges in the care of CMC (organizational policy and technology systems barriers, inadequate access to health information, and lack of partnership in care) (2) Communication solutions (shared systems that can be accessed in real-time, universal access to health information, and partnered contribution to care).

**Conclusion:**

Parents, HCPs, and teachers face multiple barriers to communication and information accessibility in their efforts to care for CMC. Parents and care providers in this study suggested potential strategies to improve communication including facilitating communication in real-time, universal access to health information and meaningful partnerships.

**Supplementary Information:**

The online version contains supplementary material available at 10.1186/s12913-021-06304-8.

## Background

Children with medical complexity (CMC) are characterized by medical fragility, technology dependency and intensive care needs [1]. Caring for CMC requires the collaboration and expertise of many healthcare providers (HCPs) spanning different disciplines, institutions, community agencies, and settings of care including the education system [1–3]. Unfortunately, when multiple care providers are involved without clear communication pathways or shared information systems, communication breakdowns are more likely [4]. Communication challenges have been identified as a significant cause of medical error and adverse events [5, 6]. Communication is a complex construct and there are multiple factors that may contribute to ineffective or failed communication including, timing, participants, information conveyed and expected outcomes [7]. These factors are magnified in the already complex nature of caring for the CMC population. In addition to the increased likelihood of communication breakdown, CMC have higher odds of experiencing in-hospital adverse events due to their complex needs when compared to children without indicators of medical complexity [8].

To date, the literature on communication challenges has focused on adult populations [9–11]. Further complexity is recognized in pediatrics given the role of family caregivers [12–15] and the multiple domains of care including education and rehabilitation [2, 3]. When caring for CMC, surveyed primary care providers describe that the most common barriers they face are the large number of specialists involved and inadequate communication between providers [16]. Emergency medicine physicians also noted communication as a major challenge when providing care to CMC [17]. Despite these findings, there remains a paucity of research exploring the communication challenges faced by *all* care providers of CMC (parents, community care providers, teachers and hospital healthcare teams).

It is crucial that we understand the communication challenges faced by parents and all care team members to find solutions that can effectively improve communication and subsequently reduce risks and gaps in the care of CMC. Thus, the objectives of this study were to explore communication challenges and solutions/recommendations from multiple perspectives including (i) parents, (ii) HCPs – hospital and community providers, and (iii) teachers of CMC, with a goal of informing patient care.

## Methods

### Study design and population

This qualitative study utilized an interpretive description (ID) design to iteratively explore communication challenges and possible solutions from the viewpoint of caregivers, HCPs and teachers of CMC. Interpretive description was the preferred methodology as it uses an inductive approach to explore a clinical phenomenon from the perspectives of those experiencing it and to apply new understandings to improve clinical care [18]. The philosophical framework of ID assumes that: 1) the researcher and the participants work together to co-create understandings of a phenomenon and 2) reality is subjective, constructed, contextual and complex [18, 19]. It aims to create and interpretive account which will guide and inform clinical practice [20]. This study is the initial component of a larger mixed methods study investigating the requirements for a novel online patient-facing platform for families and care providers of CMC to improve communication and care coordination.

Recruitment originated from the Complex Care Program at The Hospital for Sick Children (SickKids), Credit Valley Hospital (CVH), and Royal Victoria Regional Health Centre (RVH) in Ontario Canada. SickKids, CVH and RVH provide a tertiary-community approach to complex care [21] and offer a multidisciplinary complex care clinic and in-patient acute care admission services. To be eligible for the Complex Care Program, children must meet the criteria of CMC, as defined by meeting at least one criterion from each of the following conditions: technology dependence and/or users of high intensity care (e.g. mechanical ventilator, constant medical/nursing supervision), fragility (e.g. severe/life-threatening condition, an intercurrent illness causing immediate serious health risk), chronicity (condition expected to last at least six more months or life expectancy less than 6 months), and complexity (involvement of at least 5 healthcare practitioners/teams at 3 different locations or family circumstances impede their ability to provide day-to-day care of decision making for a CMC) [22]. CMC often have genetic disorders, neurologic impairment and/or cerebral palsy. Institutional research ethics approval was obtained at SickKids, CVH and RVH.

Purposive, criterion sampling guided participant selection to ensure diversity in role, practice experience, communication experience, age, ethnicity, and location to maximize the range of perspectives [23, 24]. Pediatric Nurse Practitioners (PNPs) following CMC in the Complex Care Program were requested to review their patient lists and suggest families who may be interested in participating. PNPs were felt to be the best to suggest potential parent participants as they have the most in-depth knowledge of parental experiences related to communication. Attention was paid to identify parent participants with diverse thoughts and opinions (both positive and negative) regarding communication and the idea of an online platform. Parents were eligible for inclusion if they were the primary caregiver of a CMC who had been in the complex care program for at least 3 months. Parents were excluded from participating for two primary reasons: 1) the clinical team felt research participation would be an added burden due to challenges including: end of life, acute deterioration, hospitalization, or parental physical/mental health concerns, or 2) they did not speak English. Letters were mailed to potential candidates and subsequent recruitment phone calls were made. All parents were given equal opportunity to participate or decline and participation was not influenced by PNP suggestion. Parents were contacted by a Research Assistant (RA) for recruitment not by their child’s PNP. Eligible care team members were English speaking providers both in hospital and in the community/home setting. HCPs were employees at SickKids, RVH or CVH, or provided care in the community setting (e.g. case manager, dietitian, social worker home care nurse). Teachers were eligible if they were currently teaching a CMC. All participants provided informed consent and were offered a 20$ gift card for their participation.

### Data collection

From January 2019 to May 2020, a trained Research Coordinator with qualitative experience (MB) recruited and interviewed subjects. MB obtained informed consent and individually met with participants, either in person, over the phone, or via online video conference, facilitated by GoToMeeting (Boston, MA) [25]*.* Revision of semi-structured interview guides (See the final guide in Additional file 1) occurred three times during the iterative data collection and analysis phases (which occurred concurrently) to explore further depth of emerging data. Interview questions were added as new insights were generated from earlier interviews. For example, the following questions were used in later parent interviews: 1) Have there been any situations where you did not have access to the information that you needed? How did this impact your child’s care? When you didn’t have access to the information, what did you do or who did you contact? 2) What are the barriers that prevent you from communicating using this [preferred] method? How does that impact your child’s care? and 3) Can you tell me about a time where coordinating your child’s care was challenging? Can you think of anything that would have helped in these situations? In keeping with the inductive approach of interpretive description the interview guide served to organize the interview, not to frame results or guide the data analysis. A demographic data survey was administered at the end of the study either in person or online via REDCap (Research Electronic Data Capture), a secure, web-based software platform designed to support data capture for research studies [26, 27]. Chart review was conducted for the CMC whose parents participated in the study to contextualize the patient sample.

### Data analysis

Demographic data were analyzed using descriptive statistics to summarize the characteristics of the participants in the study. All interviews were audio-recorded, de-identified and transcribed verbatim. The transcribed data was managed using QRS NVivo 12 software [28]. Consistent with ID methodology, thematic analysis was used as the qualitative data analysis approach and was completed by three team members (SA, CM, MB). Thematic analysis began following the first interview and continued concurrently with data collection to engage an iterative process. Braun and Clark’s 6 steps of thematic analysis were followed, first, analysis began with familiarization with the data done by reading and re-reading transcripts (Step 1) [29]. Following this, initial codes were generated (Step 2), team members met after every interview to review their analysis, discuss codes, merge, expand or re-code and develop a codebook. As the process evolved, codes were grouped into potential themes and themes were reviewed by comparing themes across and within participant groups (constant comparative analysis) (Steps 3 and 4) [19, 30]. Themes were continuously refined by reviewing the data within each theme. Once the final themes were reached, they were named and defined by the 3 members of the research team (Step 5). Finally, a scholarly report of the analysis was produced (Step 6).

### Methodological rigor

In order to ensure methodological rigor the following considerations were made. First, credibility was ensured through data triangulation, as the perspectives of multiple participants ranging diverse roles and multiple sites of care were garnered, and through investigator triangulation as more than 2 investigators completed coding, analysis and interpretation decisions [31]. Transferability was achieved through thick description surrounding the context of complex care [31]. Dependability and confirmability were ensured through clear description of the data collection and data analysis process, appropriate record keeping surrounding all research decision and changes made and the inclusion of direct quotations to support study findings [31, 32].

## Results

**Fig. 1:**
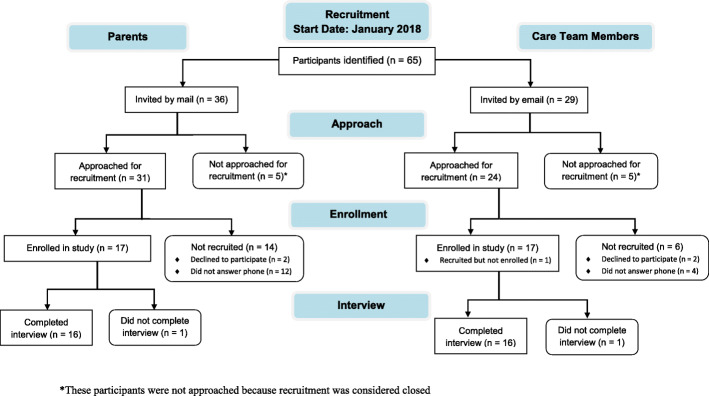
Recruitment Flowchart

Thirty-six parents and 29 care team members (HCPs and teachers) were approached to participate in the research (Fig. 1). Sixteen parents participated, 75% were mothers, and English was the primary language of 81% of participants. The CMC (*n* = 16) of the parents had a median of 11 diagnoses, 10 medications, 81% had an enteral feeding device and 63% used respiratory technology. Sixteen care team members participated, of whom 11 were HCPs and 5 were teachers. Of the HCPs 55% were based in hospital and 45% were community or home based. Descriptive data on parents and CMC (*n* = 16) and care team members (HCPs and teachers) (*n* = 16) are summarized in Tables 1 and 2.

**Table 1:** Demographic Characteristics of Parents of CMC and CMC

**Parents (*n*** **= 16)**	**n (%)**
Role	
Mother	12 (75)
Father	4 (25)
Age^a^	37 (25–47)
Primary Language	
English	13 (81)
Other	3 (19)
Marital Status	
Married or living common-law	15 (94)
Divorced	1 (6)
Education	
Completed secondary/high school	1 (6)
Some postsecondary/completed postsecondary	8 (50)
Professional/graduate degree	7 (44)
Employment	
Receiving social assistance	1 (6)
Unemployed/caregiving responsibilities	5 (31)
Employed full-time/part-time	9 (56)
Student	1 (6)
**CMC Population (*n*** **= 16)**	**Median (range)**
Number of diagnoses	11 (5–15)
Number of medications	10 (5–25)
Number of sub-specialists	8 (3–15)
Technology device, n (%)	
G/GJ tube	13 (81)
Respiratory technology	10 (63)
Vascular access	4 (25)
Mobility device	7 (44)
Communication device	3 (19)

^a^Indicates mean (range)

**Table 2:** Demographic Characteristics of Care Team Members

**Care Team (*n*** **= 16)**	**n (%)**
Role	
Nurse practitioner	2 (13)
Social worker	1 (6)
Pediatric hospitalist	1 (6)
Homecare nurse	2 (13)
Case manager	1 (6)
Dietician	1 (6)
Occupational therapist	1 (6)
Family physician	1 (6)
Emergency room physician	1 (6)
Teacher	5 (31)
Sex	
Female	16 (100)
Age^a^	43 (27–67)
**HCPs only (*n*** **= 11)**	
Primary practice setting	
Hospital based inpatient setting	6 (55)
Primary care outpatient clinic	2 (18)
Home care	3 (27)
Type of practice	
Community solo practice	1 (9)
Community group practice	1 (9)
Academic group/academic health center	4 (36)
Community hospital practice	3 (27)
Other	2 (18)
Practice fee structure	
Fee-for-service	1 (9)
Alternate payment plan	2 (18)
Salaried employee	8 (73)
Primary area of practice	
Complex Care	5 (46)
General Pediatrics	2 (18)
Other	4 (36)

^a^Indicates mean (range)

**Fig. 2:**
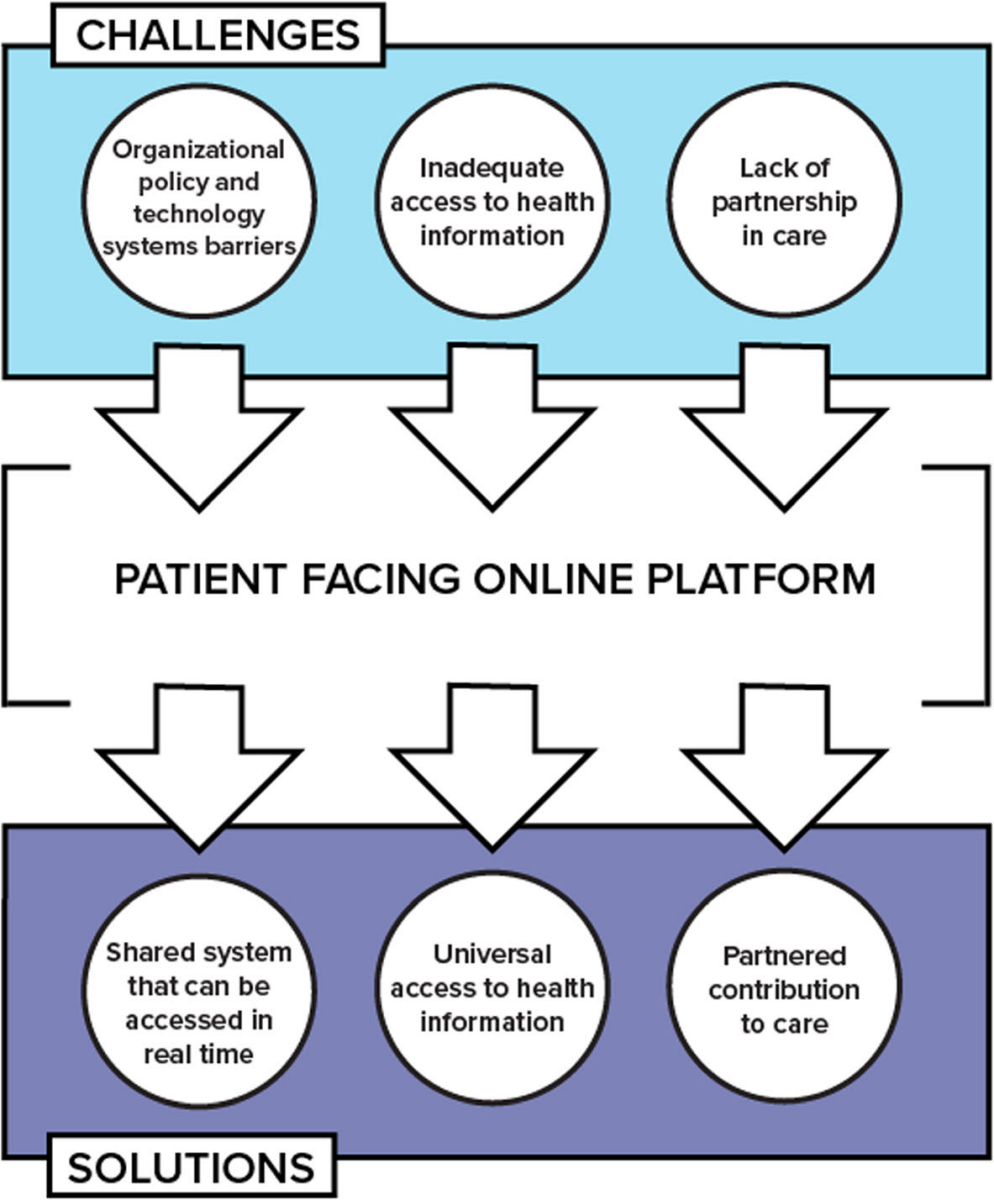
Challenges and Solutions for Communication in the Care of Children with Medical Complexity

Themes regarding communication were organized into 2 overarching themes: 1) communication challenges in the care of CMC, which outlines the current reality and 2) communication solutions in the care of CMC which demonstrates the hope for the future. These themes are further explained below and presented in Fig. 2.

### Communication challenges in the care of children with medical complexity

There were three types of communication challenges described by parents, HCPs and teachers: 1) Organizational policy and technology system barriers, 2) Inadequate access to health information and 3) Lack of partnership in clinical decision making (Table 3).

1. Organizational policy and technology systems barriers

**Table 3:** Quotes Reflecting Communication Challenges

Communication Challenges			
Theme	Parent (P) Quotes	Hospital Health Care Provider (H-HCP), Community Healthcare Provider (C-HCP) Quotes	Teacher (T) Quotes
Organizational policy and technology systems barriers	“I don’t have access to a fax machine. I used to be the one to order all of his IV medication and supplies for home and recently, [home care] said…you have to get the nurse to do it… She’s had to go to a corner store to use a fax machine. Part of me is like, you’re saying that fax is more secure, but not when you’re going to use a fax machine in a public place.” P2 “I wanna feel supported, so if I get a response to the email I’m gonna feel like I’m getting supported. If I’m not getting the response back or if I can’t send an email, I don’t feel supported.” P57 “We still use email with [health care team] once in a while, but…every time, with every new provider, I have to go in and sign permission forms, that, yes, I give them permission to use email…It takes time and energy and effort.” P55	“Parents are asking us to text, which we currently do not have a policy at all for that...so for parents the preferred method definitely is text and e-mail” H-HCP4 “A lot of the times the number that we’re given [to talk to the community physician] is the front desk number which, half the time isn’t answered. If you have the back line, it’s easy to get in touch with them. If you’re just calling the main number and having to leave a message, sometimes it can take a while to get the person you need.” H-HCP37 “Some of the community partners…may not allow any conversation via email… It takes a little while to actually obtain the consent from the family first, and then talk to the service provider at that community rehab center.” H-HCP10 “The policies around email are a little bit more strict than some other agencies. So it is challenging at times, we have to be very careful about what we share via email.” C-HCP6	“The best option would be texting, but unfortunately, we’re not really supposed to be giving out our cell phone numbers … So, that’s where my challenge kind of comes. Sometimes I go through the [school] nurse if it’s something really important, and I can’t get them [parent] by phone, then, the nurse has their work [cell] phone. So, I get that nurse to contact the parent right away.” T14 “[Medical] changes will be made… it happens via fax, …the machine isn’t working, or its busy, and that delays care at times...the fax situation is kind of ancient and I would prefer to have that come through much faster in some other digital form.” T11
Inadequate access to health information	“His medical records to me are a black box. I just started asking for copies of things, cause I have no idea how to access things [health information], or refer back to things later.. . I certainly want access to it [health information]. I wish I could get access to it [health information] easier.” P39 “I’m still trying to deal with getting his medical information. Because, [hospital] won’t give it to me unless I pay for it. I don’t feel like I need to pay to get my son’s medical records…The only way that I’m able to get them now is if I physically call each department and request for them to send it. Otherwise I need to pay to get them all on, like, a disc or paper copied, it’s like 25 cents a paper.” P56 “I can’t access her chart. I can’t see what’s being written. I should be entitled to have access to her information. But, in order to get that information, it’s hoops and hoops. It takes weeks, if not months, to get. I’ve asked, ‘can I just read her chart on the computer?’ Nope, I’m only allowed to see her chart on a computer with a physician sitting next to me. The only way we can is to go and physically pay for her chart.” P55	“I do not have access to [pediatric electronic health record], so I have nothing. I’d say, 95% of the Emergency physicians do not have [pediatric electronic health record] access. So accessing any kind of pediatric kind of healthcare information is poor.” H-HCP43 “The biggest challenge with community partners is that the record isn’t always the same. When I have a patient at [children’s developmental center], I would love to see the OT assessment that they had recently, we don’t have access to those records...So that creates a little bit of barrier on my end to have the whole picture at all times.” H-HCP2 “You’re at a standstill sometimes, so like, active issues aren’t necessarily managed because I’m waiting to get the plan, cause I don’t want to change something that might have been going well or, I don’t want to try something else, again, because I didn’t know what’s already been tried.” C-HCP41	“[Health record] goes into [principal’s] filing cabinet that’s locked up at the office. It would be super-helpful to be able to just look at it at any point in time instead of having to go, [the] ancient way, go to the filing cabinet, find the file, retrieve the piece of information.” T11
Lack of Partnership in Care	“I’m really the only one who actually knows the full picture on [son]. Everyone knows slices… I am the authoritative source for everything. Unfortunately, it doesn’t get recognized in an official capacity.” P2 “As a parent, it’s exceedingly difficult to make informed healthcare decisions for your child if you don’t know what’s being done. A test has been ordered but I don’t know about it, or a medication has been changed or discontinued. Unfortunately, it happened over and over. It was pervasive. It almost felt like a lack of respect for a parent… I want to know what’s being done.” P55 “[Communication] seems like a big favor. It doesn’t feel like communication with our specialist is something that we’re entitled to. It feels like something special that you’re granted…We felt they [health care team] were actively not listening to us, we would say, ‘A is happening,’ and they’re like, ‘no, we see B.’ You just saw him for 5 min, I spend 16 h a day with him. That was very frustrating.” P39 “Parents know their kids very well, it’s very important for the health practitioner to take them seriously, to actually listen to what they have to say, and not brush them off… It’s important… to give them the facts, so that the family can make the right decision with all the information needed.” P59	“Things like team meetings, I could attend, but I’m not necessarily included. People at [hospital] think about the people at [hospital], they don’t necessarily think about the people out in the community that are part of the team.” C-HCP41	“I have to be the initiator for some stuff, I find, they [medical team] get the information, I don’t find it’s always shared readily with me, there’s not because they’re holding back, they just gets busy.” T14 “It’s just that it doesn’t kind of come up on their radar as something that needs to be given to us so oftentimes, we miss out on paperwork that could be helpful inside their file… [easier access] would benefit the teachers and the children and the families, greatly.” T11

Parents and care team members identified a multitude of organizational policy and technology systems barriers to effective and efficient communication. Organizational policies, typically created by hospitals or homecare organizations, prohibiting e-mail and text communications due to security and privacy concerns were felt to impede communication and resulted in elaborate games of ‘telephone tag’, delays in accessing information, and negative impacts on care provision. Antiquated technology systems used for communication were identified as a barrier to communication and permeated the community, hospital and school system.

Parents expressed frustration regarding challenges they faced when trying to communicate with HCPs via email. Many parents described situations in which they were required to complete permission forms in order to be able to use email every time they met with a new provider and some parents felt a lack of support from their healthcare team in instances where they were not able to use email as a method of communication. Parents also felt that faxing was inconvenient, inaccessible and should no longer be utilized.

HCPs also described challenges when trying to communicate with other members of the healthcare team. Hospital providers noted difficulties when trying to collaborate with community providers caused by the need for parental consent before reaching out. Hospital providers also mentioned they faced challenges when collaborating with community organizations as email is often prohibited, therefore limiting direct access to community providers. This sentiment was also supported by community providers who noted challenges with email communication due to organizational policy. This lack of direct access often resulted in messages being left with office staff and communication delays. Finally, HCPs were cognizant that parents preferred texting as a method of communication however they reported being limited in this method of communication due to policy.

Like parents, teachers also expressed frustration with the health care system’s insistence on utilizing faxing instead of online communication. Teachers noted instances in which the fax machine at the school was not working or busy resulting in delays in care. Other teachers felt that the most effective way to communicate with parents would be over text, however, this is prohibited by their institution.

2) Inadequate access to health information

Inadequate access to the health information was identified as a communication challenge in caring for CMC.

Parents expressed that they did not have access to their child’s health information and some parents did not know what steps they needed to take to access their child’s past medical information other than asking for copies during appointment. Parents also discussed many instances in which they were required to pay for copies of their child’s health information and were not permitted to access these electronically. Many parents noted the inability to access their child’s health information electronically resulted in them having to wait for long periods of time before this information was made available.

Both community and hospital HCPs explained that while they could easily access information at their own organization, it was very challenging to get information from other organizations and this resulted in the inability to glean a clear clinical picture of the CMC. Sometimes HCPs noted that this inability to access all the child’s information resulted in delays in care.

Teachers expressed that the little health information the school receives is often difficult to access even within their institution and to access this information often required multiple arduous steps such as leaving the classroom, going to the principal’s office and unlocking a filing cabinet.

3) Lack of partnership in clinical decision making

Parents, HCPs and teachers also acknowledged lack of partnership in care as a communication challenge**.**

Parents reported feeling like the expert in their child’s care, but not always receiving the respect or acknowledgment of the medical team. Parents specifically expressed frustration surrounding instances in which their expertise was ignored and when decisions were made solely by the medical team without asking for or acknowledging their input. Other parents noted times where they were not involved or informed about decisions the healthcare team had made regarding their child, such as medications being changed, and tests being ordered.

Community HCPs expressed that they felt a lack of partnership with the hospital medical team. Notably, they felt that the hospital HCPs did not consider community providers to be part of the team, resulting in situations where important medical information was not shared with care team members outside of the hospital, nor was their input or expertise requested. No hospital care providers discussed a lack of partnership between themselves and community providers.

Similar to parents, teachers also felt a lack of partnership with the medical team. Some teachers described situations in which important information regarding a child’s care was not readily shared by the medical team. These situations resulted in the teachers having to seek medical information on their own and teachers felt that this information would have been beneficial to know earlier.

### Communication solutions for the care of CMC

Three core areas of improved communication opportunities were identified: 1) Shared system that can be accessed in real-time, 2) Universal access to health information, and 3) Partnered contribution to care (Table 4).

1. Shared system that can be accessed in real-time

**Table 4:** Quotes Reflecting Communication Solutions

Communication Solutions			
Theme	Parent (P) Quotes	Hospital Health Care Provider (H-HCP), Community Healthcare Provider (C-HCP) Quotes	Teacher (T) Quotes
Shared system that can be accessed in real-time	“I think [instant messaging] would allow parents to ask a question and obviously, they’re not gonna respond back ASAP, but it would at least be out there, so then you’re less likely to forget to ask that question. I find it’s [instant messaging] a lot easier, I’m able to send her photos of things right away, just to her phone, like, is this OK? She was able to respond back within 10 min and set up a plan.” P56 “If I have a simple message that I want to tell the nurse, I wouldn’t have to wait for the nurse to listen to my message, I can just say, take a look at the message portal…any medical concerns that should be mentioned to the nurse have to be privately emailed, and there’s no time for that so, it’s important to be more proactive with time. [Instant messaging] would be very helpful.” P57 “I find phone calls with updates and consultations really challenging because time is difficult. When you’re working with a part-time job and then, my daughter, whose needs are 24/7, eyes-on care, it’s really challenging to focus in on a phone call… I find it a lot easier via email, because I can do that in the evening after I’ve settled my little one into bed, and I’m waiting for the nurse to arrive.” P60	“The frustration with the physicians is not being able to contact them, which probably would be made easier with email” H-HCP37 “When I’m talking to them about the pager, and I show them something archaic from 1980, that doesn’t seem to be a problem and I wonder if, using kind of more modern technology, if people are going to expect that more instantaneous response.” H-HCP37 “People don’t want to be on the phone. They want to be able to send a quick little text and get a quick answer back…I would assume [instant messaging] would make the families feel more comfortable as well, cause they’re able to get a hold of that physician, without calling an office, waiting for a secretary.” C-HCP9 “I don’t want patients contacting me 24/7 with their questions [via text] because, I don’t have time for that. .. at least the email, I don’t have to look at it if I don’t want to, whereas my phone, like you’re constantly looking at your phone.” C-HCP41 “Well communicating as a team is always, always helpful, if we could do that on a platform. .. or be able to do something face-to-face where you don’t necessarily have to be in the home. Everybody can be at their location, would be helpful.” C-HCP7	“I shouldn’t text, they shouldn’t know my private number, but I do give them my private number. I need to be in contact with the [parents], and they don’t abuse that. Sometimes I need to call 911, when the kid’s having a seizure, so I don’t have time to go to the office [to call family]. That’s why they have my private phone number and I communicate with it when I need to.” T12 “A group message would be important if there’s multiple people concerned in the issue like the dietician, myself, the physician, and the parents.” T11
Universal access to health information	“If I’m in a situation where I don’t have my binder or it was an emergency, and I need to talk to [health care team] about something, I could pull [child’s health record] up directly on my phone, that’s helpful. Or if my husband has got [child] and something comes up, it’s shared, it’s not just in a binder at home.” P3 “I want records because records give me a lot. Let’s say if I want to apply for something from the government, I can pull that up and say, listen, this is a proof of my son’s diagnosis, from day one.” P57 “Having an ability to be able to access his medical records to show other professionals, so that we can get the help we need, I think would be super beneficial for any parent.” P56	“I just find that sometimes…you’ve got a community provider who’s got a bunch of verbal information from a family and especially if English isn’t their first language or if they’re not super historians, then you may have a community therapist that all of a sudden needs to verify some stuff.” H-HCP37 “We always hear from families about repeating their story…for the providers involved in that child’s team to be able to see that information without having to ask them for the fifteenth time, that would be beneficial.” C-HCP6 “If we could get the [hospital health record] before they come in for their first appointment, that would be helpful. It would be nice if the community pediatrician sent me the records before I was seeing the patient so I could do some background and that will make the first appointment a little bit nicer.” C-HCP41	“Having [online health information] to reference during a case conference would be amazing. I think just having that so quick and easy at my fingertips would be helpful.” T21 “It would be helpful to have previous testing results in some form… The family didn’t have a whole lot of documentation readily available and we had to request those things from the doctor. It would have been valuable to have that information ahead of time so that we could plan more effectively for care.” T11
Partnered Contribution to Care	“One time the emergency doctor who was there, said ‘before I read any of his history, you tell me what you think is going on, and I will start working on what you think is going on, and then I will look into other things.’ He said, ‘Complex Care moms know more than any of us know in the amount of every years that we went to school because they know everything about their kid’…We ended up getting the issue resolved a lot quicker.” P56 It’s more peer-to-peer. It’s working out the problem together! Not just saying ‘we’re gonna do this’…Get my opinion, get my thought process. Get me on board. Tell me what you’re thinking, so I understand why we’re doing this. It takes 5 min, but it saves you an awful lot of time down the road. P55 “She took time and talked with me and she listened to what I had to say, and didn’t push me into a decision, but basically said that you ultimately need to decide what’s best for you and the family, which is exactly what healthcare practitioners should be doing. Advising you with all the information needed for you to make a good decision.” P59	“I think it’s also nice if the family feels that they helped create the [health record] and that it really is reflective of their child and what they want people to know, not just about the medical piece.” H-HCP37 “I think it’s really important that we allow parents to be very involved in their children’s care because they are the ones primarily taking care of this child, 24/7. The doctor only sees them either when they’re sick, or when it’s a regular old check-up. I think it’s really important that parents are active within their children’s care.” C-HCP9	“We’d have to do the [care planning] together. Much like what we do with the Individual Education Plan, that has to be done with the family because you’re working towards goals together…it would be a good opportunity to work together to stay on track and come up with short- and long-term goals together.” T11 “It would be better to hear from the actual proper source [medical team] than to hear from someone else that didn’t really have the information proper.” T14

A shared system that can be accessed in real-time by everyone involved in care was identified as an ideal model of communication to link parents, community and hospital providers, and teachers.

Some parents expressed that they would appreciate a quick, effective way to communicate with their child’s HCPs and community providers outside of scheduled appointments. Parents appeared to be flexible in methods of communication; but many expressed that texting or e-mail/online communication was preferable to current modes of communication that included telephone calls or only in-person communication.

HCPs understood why families were interested in texting and email to contact their healthcare team, however, some HCPs expressed hesitation at texting and instant messaging for fear that they would be expected to return messages 24/7. HCPs expressed that easy access to each other such as through a shared email address directory or a secure portal would improve care.

Teachers felt that the best way for them to communicate with families was through texting given that their communication was often of a more urgent nature. Teacher’s also felt that the ability to quickly communicate with members of the child’s healthcare team would be beneficial.

2) Universal access to health information

Universal access to health information was acknowledged as requisite for ideal care provision by care team members and parents.

Parents felt that access to all their child’s records would make accessing their child’s health information much easier, improve information sharing, and improve overall quality and satisfaction with care. They explained that being able to view their child’s medical information could help them identify when tests and appointments needed to be scheduled so that these were not missed and would help them advocate for funding and support for their child.

Community HCPs explained that their inability to obtain information from external organizations resulted in an information gap that delayed care or resulted in potentially lower quality care. It was also felt that if all providers had access to the most up to date medical information, it may prevent the family from having to retell their story to multiple members of the care team repeatedly thus, decreasing burden on family caregivers. Hospital HCPs also noted times in which their community counterparts did not receive accurate medical information, which caused confusion, something that could be avoided if there had been universal access to the child’s health information.

Teachers echoed the feelings of HCPs noting that being able to access their student’s health information on their own, without having to ask parents or ask the medical team would beneficial. Specifically, teachers described how universal access would enable them to better plan for ways in which they could support the CMC in the classroom.

3) Partnered contribution to care

Partnered contribution to care was recognized as a way to facilitate improved care provision.

Parents described instances in which they felt accepted and listened to as true members of the healthcare team and noted that care was provided more quickly and efficiently as a result.

Both community and hospital HCPs agreed that it is extremely important to allow families to have increased involvement and that it could be helpful in providing a more holistic picture of the child and family to the healthcare team. Some HCPs discussed the possibility of parents contributing directly to their child’s health record, noting that this was another way in which partnership could be promoted.

Teachers were interested in partnering with parents to create school specific plans of care, an activity that they felt would foster improved care provision and goal setting. Teachers also felt that direct partnership between the education and medical teams would be beneficial in allowing them to obtain the most accurate information about the CMC and their required care.

## Discussion

This is the first qualitative study to explore communication challenges experienced amongst all domains of care providers for CMC (healthcare, family caregiver, education). Care providers of CMC face organizational policy and technology system barriers that impair their ability to communicate and access information effectively therefore impairing the opportunity for partnership and increasing fragmented care. Our results highlight the need for improved communication methods allowing for shared systems that can be accessed in real-time by all team members, including the family, universal access to health information and improved partnership between the family, teachers, community and hospital HCPs. It is important to note that our findings clearly support the need for improved family-centered care (FCC). FCC is an approach to healthcare decision making that is based in partnership between families and HCPs [33]. There are several principles involved in FCC, two of which include information sharing and partnership and collaboration, both of which align very clearly with the communication solutions of a shared system accessible in real-time, universal access to health information and improved partnership [33]. We posit that implementing methods to improve communication in real-time and universal access to health information may provide greater opportunities for improved partnership between all members of the healthcare team and ultimately improved FCC.

It is well known that communication breakdown between health care team members is a leading cause of medical error and patient harm [5, 34, 35]. Optimal communication and teamwork are essential in supporting a culture of safety in the delivery of patient care [34]. The majority of research to date has been conducted in the in-patient hospital setting and highlights structured communication techniques (e.g. briefing and closed communication loops) as the main strategy to improve communication and patient safety [36, 37]. Studies in the primary care and emergency department settings focused on challenges faced when caring for CMC and noted communication among providers as a pervasive barrier [16, 17]. Some studies have investigated communication challenges faced by pediatricians and teachers. Pediatrician barriers to communication included: lack of time, inaccessible school personnel and not knowing with whom to collaborate while teachers noted not knowing they could communicate with pediatricians and privacy concerns [38, 39]. Our findings differ from the literature in that identified communication challenges are specific to the care of CMC and were described by many different care providers, across many levels of care, including hospitals, community health care, and the school system. Namely, institutional policies limiting modern communication methods and siloed information systems contributed greatly to the communication challenges faced by care team members of CMC. Organizational policies prohibiting text and email communication due to security and privacy concerns force parents and HCPs to rely primarily by telephone communication which is inherently flawed. Busy care schedules do not allow parents or HCPs to be available to take a telephone call at all hours of the day which results in multiple messages left and calls being unsuccessfully returned – delaying communication. This theme of limited access is also prevalent when it relates to health information. Privacy concerns result in siloed health records that are only visible to a small group of HCPs do not allow for open exchange of information between parents and providers or amongst wider groups of providers at different institutions. This can result in substandard care provision that is not based on the most recent patient information. Participants expressed the need for realistic updates to antiquated hospital, health agency, and school policies to allow for open communication using modern methods such as e-mail and texting [40–42].

In addition to being at increased risk of communication breakdown, CMC are also at higher risk for safety issues and care gaps simply due to the complexity of their medical needs [43]. Communication challenges experienced by multiple care providers [44] in different organizations [45] increases the likelihood that CMC will experience gaps in care or adverse events [4, 46, 47]. Parents and care team members in our study agreed that siloed information systems made accessing a child’s health information time consuming, tedious and expensive, and often resulted in missing information. Breakdowns in communication can occur because the fragmented healthcare system makes it virtually impossible for HCPs to access information from outside of their institution, in turn making it difficult to ensure that medical information is accurate and up to date [1, 4, 48]. When HCPs have incomplete or outdated patient information it can increase the chance of families experiencing gaps in care [4, 48]. Care gaps have been shown to be especially pervasive among CMC, with as close to 50% of families of CMC experiencing at least one unmet medical service need [14]. We suggest that addressing the identified communication challenges by updating policies to facilitate shared communication systems that can be accessed in real-time between all care team members (e.g. parent, homecare nurse, teacher, PCP and sub-specialist) and universal access to health information are imperative to improve the safety of CMC.

Parents in our study felt a lack of partnership, as some described not being acknowledged for their expertise and not able to contribute to their child’s plan of care. True collaboration and partnership require parents to be valued as key members of the healthcare team. Previous research in the neonatal intensive care unit (NICU) has shown that the family-integrated care (FICare) framework, in which parents are integrated, accepted and recognized as key members of the care team, improves parental confidence and parent-HCP communication [49]. The success of FICare highlights the importance of shared decision making (SDM) and family partnership within the clinical setting. SDM is a principle of communication [50] that forms the basis of patient and FCC [51] and occurs when parents and HCPs collaborate in all phases of the decision-making process to agree upon a treatment plan together [51, 52]. As CMC are at high risk of experiencing care gaps and patient safety events, SDM is essential in the care of CMC as it provides the opportunity for longitudinal alignment of care decisions [53]. Further, improving SDM may improve parent-provider communication as it allows space for discussion surrounding the parent’s understanding, values and self-efficacy [54, 55]. Our findings are in accordance with previous research demonstrating that parents of children with chronic medical conditions want to be involved in SDM as active partners in care [56–58], despite this, families of CMC have a lower likelihood of actually being involved in SDM [53]. Our results also demonstrate that HCPs in the community felt a lack of partnership with the hospital care providers, this is supported by the literature which has shown that direct communication between hospital and primary care physicians is infrequent [9]. Our results, along with the literature, clearly demonstrate the need for a culture shift within the healthcare and education fields to further support partnership and SDM between parents, community care providers, teachers and hospital HCPs.

The limitations to this study are noted. Parents of CMC were predominantly mothers and care team member participants were all women. Therefore, our results may not clearly reflect the experiences of fathers or male care team members. The bias towards female care team members has been previously demonstrated in the pediatric setting [59]. Due to a lack of access to interpreter services, parent participants were English-speaking, limiting the ability for our results to represent the experiences of more linguistically diverse families. Finally, this study is limited in scope as it reflects experiences navigating the healthcare and school systems in Ontario, Canada, therefore the results may not be representative of other jurisdictions.

Further study is needed to investigate novel ways to bridge the multiple communication challenges identified by this study. Evaluation of outdated communication policies and implementation of more modern-day communication methods such as secure messaging is a simple step that has proven successful in some hospital and community settings [60–65]. Integration of information systems across all agencies is needed. Novel methods such as patient-facing platforms have proven beneficial to improve communication, health outcomes and access to information in adult care [66–69]. Patient-facing platforms are internet-based websites or phone applications with a variety of capabilities such as secure messaging between patients and HCPs, and provide patient access to their own medical records. In pediatrics, patient-facing platforms have supported secure messaging, post-discharge follow up, and have been suggested as a solution to improve access to information in the care of CMC [70–72]. Therefore, future research endeavors are needed to understand the role of shared communication systems that can be accessed in real-time, universal access to health information and their potential to positively impact partnership and ultimately improve communication and FCC.

## Conclusion

Multiple aspects of communication in the care of CMC pose a challenge to parents, HCPs, community providers and teachers. An overhaul of antiquated policies to allow for open communication and information exchange using common, accessible methods such as e-mail and texting is essential. Access to systems that facilitate real-time communication, access to *all* health records from multiple institutions for HCPs and parents, and improved partnership between all those caring for CMC, are long overdue and needed to provide timely, safe and family centered care. The findings from this study can be used to inform the development of a patient-facing platform to improve communication and collaboration in the care of CMC.

## Supplementary Information

**Additional file 1:.** Semi-Structured Interview guides.

## Data Availability

The datasets used and/or analysed during the current study are available from the corresponding author on reasonable request.

## Abbreviations

CMC: Children with Medical Complexity
HCP: Health Care Provider
RVH: Royal Victoria Regional Health Centre
CVH: Credit Valley Hospital
SickKids: The Hospital for Sick Children
FCC: Family Centered Care
