# Supplementary material for: Perspectives on team communication challenges in caring for children with medical complexity
Source: BMC Health Serv Res. 2021 Apr 1;21:300. doi: 10.1186/s12913-021-06304-8 (PMC8015748; doi:10.1186/s12913-021-06304-8)
Supplement: Supplementary file 1 — Additional file 1:. Semi-Structured Interview guides. [file 12913_2021_6304_MOESM1_ESM.doc]

**STUDY A – Parent Semi-Structured Interview Guide**

**Note:** Specific questions may be modified from interview to interview depending on participant responses and based on feedback from previous interviews.

**Introduction**

Thank you for taking the time to participate in this study! Today, we’ll be talking about your experience as a parent of a child with complex care needs to help us learn how we can work together to improve communication and care coordination.

There are two questionnaires for you to complete and then we will begin the interview part of the visit. The interview will ask general questions about you and your child. If you are interested, I can show you some screenshots of the online platform that we have developed. Your open and honest feedback will help us understand some of the current challenges and modify the current platform to be more reflective of what parents/caregivers want. The visit will take approximately an hour and a half to complete.

All information collected in the study will be kept confidential. All personal identifying information will be removed from the data and study data will be stored in a secure, locked location. Only members of the research team will have access to the data. Following completion of the study, the data will be kept for seven years and then destroyed as required by Sick Kids policy.

Just a reminder that this interview will be audio recorded and only members of the research team and a professional transcriptionist will hear the recording. If you do not wish to be audio recorded, you may withdraw from the study. Your participation in this interview is voluntary and you can decide not to answer questions or can withdraw from the study at any time, even once we begin the interview. Continuing with the interview implies consent to be a part of the study. Please feel free to ask any questions at any point during the visit.

Do you have any questions before we begin?

1. Can you start off by telling me about your child? Who he/she is as a person and a little bit about his/her medical challenges?

**Communication with care team**

We’ve heard from parents that caring for a child with medical complexity involves communicating with a number of care providers including health care providers in the hospital and community, as well teachers, to coordinate your child’s care. We want to hear about what communication with all these care team members has been like for you.

1. When caring for your child, who do you usually communicate with (i.e. teacher, home care nurse, complex care team, school nurse, pediatrician)? How often do you communicate with them and how do you communicate with them (i.e. in-person, email, phone)? Do you find that works well? What does/doesn’t work well about it? What do you think would make communication better for you?
2. We understand that there are limitations to how you can communicate with your health care team due to privacy concerns related to parents and health care providers sharing personal health information through non-secure methods. For example, communication outside of appointments is generally limited to phone and email rather than other communication methods such as texting. How would you want to communicate with your HCPs outside of appointments if privacy was not an issue? How do you think communicating in this way would help you? Why is this important for you? What are the barriers that prevent you from communicating using this method? How does that impact your child’s care?
3. Would you use a secure messaging system (similar to Facebook chat) instead of how you currently communicate? Why is having a secure messaging system important for you? How do you think this would change your experience communicating with others? Why wouldn’t you use a secure messaging system?
   1. If you think about using a messaging system to communicate with your HCPs, what would you need out of that system? *For example, this could be the ability to group chat, include photos or links, communication be integrated into EMR (or not).*
   2. Would you feel comfortable having these conversations included in your child’s electronic medical record? This means that other HCPs would be able to see the conversations.
4. Can you think of any situations where you had issues communicating with your child’s care team? This could include hospital (specialists, complex care, inpatient team) or community providers (home care nurses, teachers, OT, PT). What do you think would have helped in this situation?
5. Can you think of any positive experiences communicating with different care team members? What made these experiences better for you?

**Coordination of care and roles**

We’ve heard from families that they are often responsible for updating care team members on their child’s test results or changes to their child’s health condition. We’d like to learn more about your experience coordinating your child’s care and the role you want to have in your child’s care.

1. What has your experience been like coordinating your child’s care? This includes in the hospital and community with both healthcare and non-healthcare providers. Has anything made coordinating your child’s care better or easier?
2. Can you tell me about a time where coordinating your child’s care was challenging? Can you think of anything that would have helped in these situations?
3. What role do you want to have in your child’s care? Do you want to be the coordinator? How much coordination do you want to do? Would you like to be the custodian of your child’s health information? *This includes choosing who is able to access information.*
4. Do you see yourself as a partner in your child’s care? What has made you feel like a partner in care? What do you think could be done differently to make you an active partner in your child’s care?
5. We are currently evaluating the idea of an online care plan that parents can access and edit. How do you feel about contributing to your child’s medical information? Would you want to update your child’s medical care plan yourself? Are there parts that you would not feel comfortable editing?

**Accessing health information**

Families have told us that they do not have access to all their child’s medical records and this has made coordinating their child’s care more difficult. We know that there are some platforms, like MyChart, that give you limited access to your child’s health information.

1. Can you tell me about your experience accessing your child’s health information?
   1. Have there been any situations where you did not have access to the information that you needed? How did this impact your child’s care? When you didn’t have access to the information, what did you do or who did you contact?
   2. When you had access to the information, how did this help you or your child’s care?
   3. What information do you want access to? Would you want to access the full chart or only certain parts? How would you like to have access to this information (i.e. online platform, through email, paper-based, etc.)? How do you think this would help you?
2. Do you find that your child’s care team has access to the same up-to-date medical information (i.e. home care, school, OT, in-hospital team)? If not, how has this impacted your child’s care? What have you needed to do in those situations?
3. When you go to an appointment at SickKids, do you find that the provider has the information that they need?
4. When you go to an appointment at another hospital (such as an ER visit), do you find the provider has the information that they need?
5. When you go to a community-based appointment such as with your pediatrician, family doctor, or allied health, do you find that the provider has the information that they need?

Those are all the questions that I have for you today! Thank you so much for participating in our research study. We really appreciate your input and we hope to use it to create the best possible tool.

Now I will turn off the audio recorder.

**STUDY A – HCP Semi-Structured Interview Guide**

**Note:** Specific questions may be modified from interview to interview depending on participant responses and based on feedback from previous interviews.

**Introduction**

Thank you for taking the time to participate in this study! Today, we’ll be talking about your experience as a health care provider for children with medical complexity to help us learn how we can work together to improve communication and care coordination.

I will ask you questions throughout the interview about your experience communicating with patients and families, as well as other health care providers. If certain questions are not relevant to your role, feel free to say they are not applicable. Your open and honest feedback will help us understand some of the current challenges that you and families are facing. We have two short questionnaires for you to complete after the interview. The visit will take approximately an hour to complete.

All information that is collected in the study will be kept confidential. All personal identifying information will be removed from the data and study data will be stored in a secure, locked location. Only members of the research team will have access to the data. Following completion of the study, the data will be kept for seven years and then destroyed as required by Sick Kids policy.

Just a reminder that this interview will be audio recorded and only members of the research team and a professional transcriptionist will hear the recording. If you do not wish to be audio recorded, you may withdraw from the study. Your participation in this interview is voluntary and you can decide not to answer questions or can withdraw from the study at any time. Continuing with the interview implies consent to be a part of the study. Please feel free to ask any questions at any point during the visit.

Do you have any questions before we begin?

1. This will be in the demographics questionnaire as well, but just to give context for the interview, what’s your role? Where do you work? Who do you work with?

**Communication with patients and families**

1. How often do you find yourself communicating with the families of complex care patients? How is this usually done? Do you find that works well? What do you like about that form of communication? What are some of the challenges with these methods?
2. We understand that there are limitations to how you can communicate with your patients due to privacy concerns. For example, communication outside of appointments is generally limited to phone and email rather than other communication methods such as texting. If privacy was not an issue, how would you want to communicate with families? How do you think communicating in this way would help you and families? Why is this important to you? Are there any barriers right now that prevent you from communicating in this way? How does that impact care?
3. We are evaluating the idea of an instant messaging system for patients and their care team. Patients would have access to the instant messaging system at all times. Do you have any experience using an instant communication system with your patients? Do you have any fears about instant messaging? How would you set boundaries with instant messaging? What would an instant messaging system need to have to make it useful for you? *This could include group chat function, out of office, read responses, send files.*
4. Can you tell me briefly about any challenges that you face related to communication for your complex care patients? What do you feel like is currently missing? What would make these situations better? *This could be communication with other health care providers, community members, family, getting full medical history, etc.*
5. What do you find families expect from you regarding communication? How do you manage these expectations?
6. How do you set boundaries with your patients for communication? Have you had any issues with boundaries with your families?
7. Can you tell me about any situations where you had issues communicating with families? What would have helped in that situation?

**Communication with health care providers**

1. What does communication look like with other health care providers across different settings (i.e. community care, school, rehab centers, in-hospital)? Who do you communicate with? How is this usually done? What do you like about that form of communication? What are the challenges? What are the barriers?
2. A lot of complex care patients have allied health professionals in different settings. How do you effectively communicate with all these different parties? Are there any challenges with this? What could make it better?
3. Can you think of any situations where you had issues communicating with other providers? How did this impact the patient and family? What could have helped in this situation?

**Access to information**

1. What has your experience been like accessing health information from providers in different settings (i.e. community allied health professionals, other hospitals)? What does this process usually look like? What are some of the challenges with the current process or system?
2. What information can you currently access? What information do you want access to that you don’t currently have? Why don’t you have access to this information? How would you want access to this information? How do you think this would help you?
3. Can you tell me about a situation where care was affected because you or another health care provider did not have access to the information that you needed? How do you think the situation could have been improved?
4. How do you feel about parents having access and ownership over their children’s medical information? How do you see this affecting your care or influencing your role? How do you feel about parents contributing to their child’s medical documentation?
5. Technology is advancing rapidly and impacting the world we live in. However, the health care system has been very stagnant with respect to technology. Are there any challenges in caring for your Complex Care patients that you think could be improved by technology? *For example, patients receiving text reminders about appointments or the option to do an appointment virtually.*

**Roles**

1. How do you set expectations with families about your role? Do you find that you are ever asked by families to do anything beyond your role?
2. How do you set expectations with other health care providers about your role? Do you find that you are ever asked to do anything beyond your role?

Those are all the questions that I have for you today! Thank you so much for participating in our research study. We really appreciate your input and we hope to use it to create the best tool possible.

Now I will turn off the audio recorder.
